# Cancer Diagnosis through SERS and Other Related Techniques

**DOI:** 10.3390/ijms21062253

**Published:** 2020-03-24

**Authors:** Maria Blanco-Formoso, Ramon A. Alvarez-Puebla

**Affiliations:** 1 Department of Physical Chemistry and EMaS, Universitat Rovira i Virgili, 43007 Tarragona, Spain; 2 ICREA, Passeig Lluís Companys 23, 08010 Barcelona, Spain

**Keywords:** cancer, diagnosis, liquid biopsy, miRNA, circulating tumor cells, plasmonic nanoparticles, SERS, SPR, DLS, TIRF

## Abstract

Cancer heterogeneity increasingly requires ultrasensitive techniques that allow early diagnosis for personalized treatment. In addition, they should preferably be non-invasive tools that do not damage surrounding tissues or contribute to body toxicity. In this context, liquid biopsy of biological samples such as urine, blood, or saliva represents an ideal approximation of what is happening in real time in the affected tissues. Plasmonic nanoparticles are emerging as an alternative or complement to current diagnostic techniques, being able to detect and quantify novel biomarkers such as specific peptides and proteins, microRNA, circulating tumor DNA and cells, and exosomes. Here, we review the latest ideas focusing on the use of plasmonic nanoparticles in coded and label-free surface-enhanced Raman scattering (SERS) spectroscopy. Moreover, surface plasmon resonance (SPR) spectroscopy, colorimetric assays, dynamic light scattering (DLS) spectroscopy, mass spectrometry or total internal reflection fluorescence (TIRF) microscopy among others are briefly examined in order to highlight the potential and versatility of plasmonics.

## 1. Introduction

According to the WHO (World Health Organization), cancer is the second leading cause of death worldwide (following cardiovascular disease), causing around 9 million deaths per year [1]. Approximately, 1 in 6 deaths in the world is due to this multifactorial disease characterized by the uncontrollable growth of cells: anomalous ones deepen in healthy tissues invading adjacent parts or spreading to other organs, in the process known as metastasis. 

The detection of this disease in an advanced stage with the consequent lack of treatment is a frequent problem that leads to a substantial and increasing economic impact of cancer [2]. For this reason, one of the vital challenges in novel medicine is to detect malignant tumors in early stages, even the pre-cancerous lessons. Moreover, focusing on a personalized diagnosis is crucial as the same cancer can vary from person to person (even cells in the same tumor present heterogeneity due to the environmental epigenetic changes affecting both phenotype and function) [3]. Both approximations to the accurate and early diagnosis give rise to a rapid treatment, which would improve the prognostic of cancer patients, with less morbidity, high probability of surviving, and also better quality of life not only for individual patients but also for caregivers and community in general, having a beneficial impact on social costs.

Classical techniques for cancer diagnosis comprise anatomic imaging tools like X-ray imaging and computerized tomography scan (CT) or positron emission tomography (PET) for physiological and metabolic information, respectively [4]. The disadvantages of the above-mentioned techniques are the submission of the patient’s body to ionizing radiation [5]. Those that do not belong to radiological tests, like magnetic resonance imaging (MRI) [6] must be used under heavy magnetic fields during the exploration, which is incompatible with patients using pacemakers or metal implants. Further, these techniques are used sometimes in combination with contrast agents, responsible for causing secondary effects like allergies or posing risks for patients with renal impairment. Another alternative is the use of ultrasounds, which produce sonograms in real-time, but with the inherent limitation of low-resolution [7]. 

**Figure 1:**
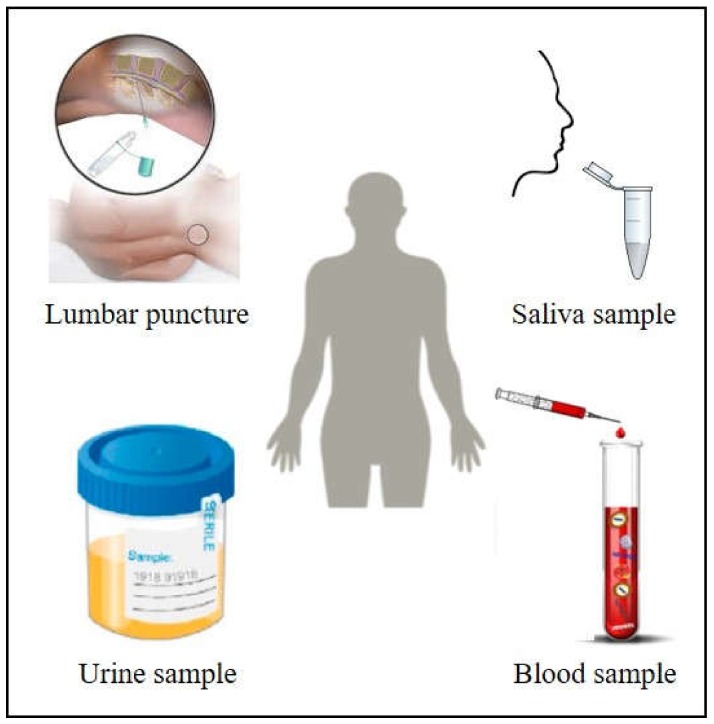
Body fluids as blood, saliva, urine or cerebrospinal fluid are excellent candidates to perform non-invasive cancer diagnosis based on plasmonic sensors. Adapted from [9] with permission. Copyright Frontiers 2019. Adapted from [10] with permission. Copyright American Chemical Society 2018. Adapted from [11] with permission. Copyright Wikipedia 2014.

Nowadays, the alternative that is increasingly present in diagnosis and monitoring of cancer is the employment of plasmonic particles as improved devices. This approach, here reviewed, is based on optical methods, which present notable benefits in comparison with current techniques in order to complement or substitute them, as they are fast, accurate, precise and with high spatial resolution [8]. They overcome the disadvantages of routine methods by not using ionizing radiation, which makes them non-invasive methods. Indeed, the use of body fluids like blood, urine, saliva, cerebrospinal fluid or breast milk (Figure 1) mirrors in a feasible and non-invasive manner what is happening inside the affected body tissues, opening the doors for a real alternative to standard tissue biopsy, as liquid biopsy provides faster results with the ability to establish the risk of metastasis and monitor cancer evolution in a more frequent, cheaper, and definitely more efficient manner.

**Figure 2:**
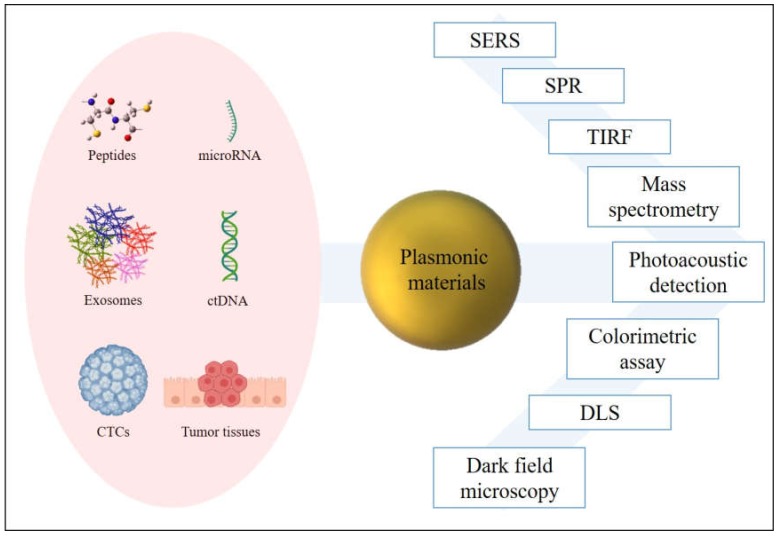
Plasmonic materials improve the detection of cancer biomarkers through several techniques.

In those fluids, diverse materials susceptible to carrying cancer information are released becoming potential excellent biomarkers (Figure 2). Most studied are proteins or peptides, genetic material, or circulating tumor cells (CTCs) detached from tissues. In this framework, microRNAs (miRNAs) have obtained recognition as cancer biomarkers due to their evidenced roll in tumor progress and metastasis [12,13], although their short length, low concentration in body fluids, and similitude between members of miRNA family make measurement a challenge with conventional methods [14]. Circulating tumor DNA (ctDNA) handle the tumor-specific sequence modifications, being useful as prognosis agent [15,16]. Indeed, its short half-life (less than 2 h in blood), ensures a real-time tracking of the tumor evolution [17]. Exosomes created after vesiculation of late endosomes and released via explicit fusion with the external membrane of the cell contain valuable information of the cell of origin, emerging as potential biomarkers in the last decades [18,19]. CTCs and tissue examination carry really valuable information, because of the high variability of phenotypes not only between individuals but also within an individual tumor lesion. Indeed, CTCs play a critical course of metastasis [20,21].

The optical properties of nanostructured metallic particles are derived from the collective excitation of their conduction electrons with light [22,23]. A small metallic particle (≤ 100 nm), illuminated with light, generates a strong electric field at the surface due to the generation of dipoles (and/or multipoles) inside the nanoparticle. Resonance occurs when polarizability experiences a maximum, which physically results in the coherent and collective oscillation of the conduction band electrons. Because the electron cloud is confined in the surface of the nanoparticle, this effect is called localized surface plasmon resonance (LSPR) [24,25]. LSPR has two transcendental consequences: electromagnetic fields at the nanostructure surface are greatly enhanced, and the particles’ absorption and scattering have a maximum at the plasmon resonant frequency [26]. LSPR depends on the size, geometry, and composition of the nanostructure, geometry being the most important. This versatile effect is one of the reasons why metals have so much potential for nano-optics. Besides, electromagnetic fields can have even more specific shapes such as tips [27] or the so-called hot spots [28]. Attending to the material, noble metals, gold and silver have attracted huge interest among the plasmonic nanoparticles. However, although Ag outperforms Au as enhancer, usually gold is preferred because of its greater chemical stability and biocompatibility with minimum toxicity for biological applications [25]. 

At this point, plasmonic materials present a multitude of potential applications in cancer diagnosis. Here, we review the different approaches of diagnostic nanodevices with ultrasensitive features that complement and improve current methodologies in cancer diagnosis.

## 2. SERS Methods

Surface-enhanced Raman scattering (SERS) is a recognized spectroscopic technique with ultrasensitive spatial resolution, which outperforms classical techniques such fluorescence: it provides narrow bands of 0.1 nm in comparison with 20–80 nm bandwidth for fluorescence, being suitable for multiplexing detection in the same sample allowing the identification of several targets in simple assays. Obtaining a high volume of valuable data in parallel saves time and costs to clinicians [29]. Another advantage over fluorescence, is that plasmonic labels are not vulnerable of photobleaching, and the limit of detection (LOD) achieved with SERS is around three orders of magnitude smaller than LOD obtained with fluorescence [30,31]. SERS combines Raman spectroscopy, which provides specific structural information based on molecules’ vibration modes, with the capacity of signal-amplification of plasmonic nanoparticles. The plasmonic effect enhances Raman scattering intensity through the electromagnetic mechanism (EM), derived eminently from the plasmonic nanostructure and the excitation light, with additional contribution from the chemical effect (CT), due to the charge-transfer between the nanostructure and the molecule. EM can be explained as the contribution of the enhancement of the incident field at a certain point on the surface where the analyte is positioned and the enhancement of the re-emitted Stokes scattering by the molecule. CT mechanism occurs through the formation of a new analyte-metal surface complex, where the electronic properties of the attached molecule are distorted enabling new transitions within the complex. Modified polarizability of the analyte leads to an enhancement of the Raman cross-section. Both mechanisms occur together adding their effects [25]. Since each molecule has a unique Raman spectrum, it has become a fingerprint that can be used for the characterization of even single tumor cells. Furthermore, it is a non-destructive technique that can be applied to a little amount of solid and liquid samples requiring minimum preparation. 

Among the SERS nanosensors developed in the last decades to diagnose cancer, some approaches used bare plasmonic nanoparticles whereas most of them use a Raman probe, whose spectral variations in shift or intensity will be monitored. Table 1 summarizes the latest advances in both approximations.

### 2.1. Label-Free SERS

**Figure 3:**
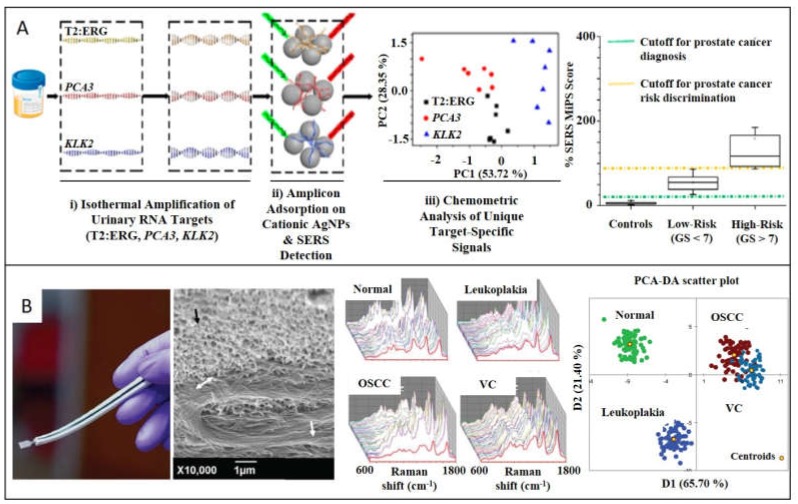
SERS detection of cancer biomarkers through label-free SERS nanoparticles. (**A**) Urine samples from patients with prostate cancer were collected to further isothermally amplify RNA targets (T2:ERG, PCA3, and KLK2) for being detected by SERS substrates. Adapted with permission from [10]. Copyright American Chemical Society 2018. (**B**) Dual-function catheter to take oral cancer samples and act as SERS substrate in situ (interface of tissue/nanostructure is signaled in the SEM image by the double-headed arrows. Collected spectra are averaged, scored, and grouped to discern between oral squamous cell carcinoma, verrucous carcinoma, and premalignant leukoplakia. This allows the diagnosis of healthy, premalignant and malignant oral cancer. Adapted with permission from [32]. Copyright Wiley 2019.

The nonspecificity and high background habitually difficult the assignment of Raman bands making this approach less popular. On the other hand, direct measurements are advantageous for their fast, cost-effective and easy-to-use performance. Furthermore, the lack of accuracy can be overcome through amplification processes of the target, suitable in genetic material detection because of the several techniques for replicate the nucleic acid biomarkers that exist [44]. Specifically, for nucleic acid sequences, direct adsorption onto plasmonic materials through the negatively charged phosphate groups enables direct label-free readout strategies saving time-consuming labeling [45,46]. Koo et al. designed a label-free SERS diagnostic technology for differentiating between high- and low-risk prostate cancer (PC) (Figure 3A) [10]. T2:ERG, PCA3, and KLK2 miRNA (PC specific targets) from urine samples are amplified to enhance SERS intensity, through an isothermal process, which results in the stabilization of native miRNA in double-stranded DNA. After mixing the targets with silver nanoparticles (AgNPs) of 40 nm in size, SERS spectra were collected. Differences among Raman patterns of three targets spectra were identified and clustered into three groups through principal component analysis (PCA). With this statistical method, the high number of variables is reduced to a small set of dominant dimensions to further stablish a risk stratification scoring system by analyzing a training cohort (*n* = 80, 20 healthy patients, 60 PC samples divided into low- and high-risk). This methodology provides results in 90 min, shortening alternative methods, which habitually take more than 4 h. Lin et al. developed a similar label-free SERS-based dispositive to detect blood circulating DNA (cDNA) of nasopharyngeal cancer (NPC) [34]. In this approach, the whole cDNA is considered a biomarker, saving even more time compared to alternative methods that isolate determined targets. It consists in extracting cDNA from NPC patients (*n* = 120) and control subjects (*n* = 120), to further mix with AgNPs of 35 nm in size. Although as-synthesized AgNPs are stabilized by chloride anions, DNA divalent phosphate backbones displace mentioned monovalent anions. After 3h of incubation, one drop of MgSO_4_ is added in order to aggregate the sample causing hot spots to increase the SERS enhancement. Differences between SERS spectra of control and NPC group were obtained after multivariate algorithm analysis based on PCA combined with linear discriminant analysis (LDA) in order to identify the significant variables, achieving diagnostic sensitivity of 83.3% and specificity of 82.5% among NPC and control group.

Following the analogous strategy, Carmicheal et al. [33] designed a label-free substrate for early diagnose of pancreatic cancer based on the identification of exosomes extracted from serum of healthy donors (*n* = 10) and pancreatic cancer (PaC) patients (*n* = 10). Plasmonic material consists of gold slides, and positively charged AuNPs of 10 nm are deposited onto it to caption negatively charged exosomes from C18/HPAF, MiaPaCa, and HPDE pancreatic cancer cells. Collection of SERS spectra was then submitted to PCA algorithm, providing good results in terms of sensitivity and specificity. Previous studies developed by Park et al. focused also their efforts on discerning among exosomes originated from healthy and lung cancer cells by SERS-PCA [47]. Stremersch et al. differentiated exosomes from melanoma and healthy red blood cells by developing SERS-partial least squares discriminant analysis [48]. Moving to tissue samples as biomarker, Girish et al. fabricated a catheter to classify and stablish grades (healthy, premalignant, and malignant) of oral cancer by testing oral squamous cell carcinoma, verrucous carcinoma, and premalignant leukoplakia (Figure 3B) [32]. The sensor is made by a TiO_2_ leaf-like nanostructure equipped with AgNPs of 30 nm in size, which serves to extract cancerous tissue from mouth and directly make the SERS measurements. From averaged spectra from the four different groups, discriminant function scores were obtained. PCA-DA cross-validation was carried out with an accuracy of 97.24%. It is worth noting that this patient exam can be developed in around 30 min, outperforming conventional histopathology process time based on invasive biopsy, which includes surgery, cryogenization, and biochemistry techniques. Previous studies of label-free SERS in cancer tissues are reported in literature [49,50].

### 2.2. Encoded-SERS

**Figure 4:**
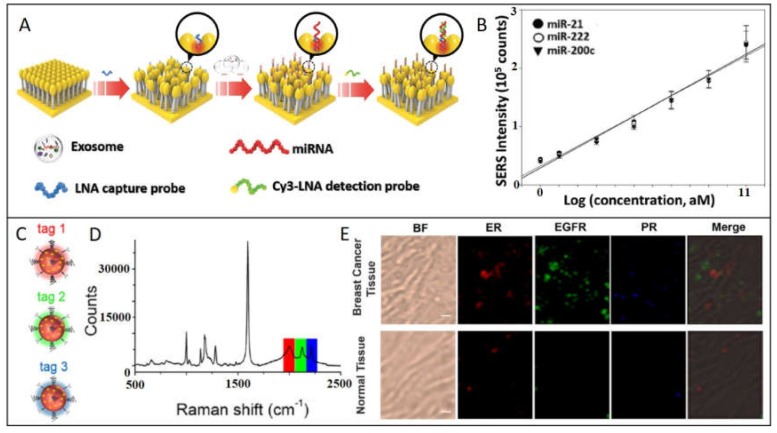
SERS detection of cancer biomarkers using SERS-encoded nanoparticles. (**A**) Schematic depiction of exosomal miRNA detection through the head-flocked gold nanopillars SERS sensor. locked nucleic acid (LNA) probes are attached to the AuNPs to further immobilize target miRNAs (miR-21, miR-222, and miR-200c) and Cy3-labeled LNA detection probes, resulting in the sandwich structure responsible of the strong SERS signal. (**B**) Linear regression analysis showing the relationship between Cy3 SERS intensity at 1150 cm^−1^ and target miRNA concentration. Adapted with permission from [39]. Copyright Wiley 2019. (**C**) Au NPs 60 nm in size are functionalized with three different SERS tags/antibodies: Tag 1 (4-ethynyl-bipheny/ER), Tag 2 (phenylthiocyanate/EGFR), Tag 3 (4-(phenylethynyl)aniline/PR) (**D**) The SERS spectrum of the mixture of tag1, tag2, and tag3. (**E**) Multiplex profiling of ER, EGFR, and PR expression in breast cancer tissue and normal tissue sections after treatment with a mixture of tag 1, tag 2, and tag 3. Adapted with permission from [40]. Copyright Cancer Nanotheranostics 2019.

A refined SERS-based strategy consists in synthesizing hybrid nanostructures composed by the plasmonic nanoparticle functionalized with a molecule with high Raman cross-section (named reporter or probe); then, plasmonic nanoparticle can be protected with an out layer of polymer or silica. These SERS-encoded particles (SEPs), can be functionalized with specific antibodies or proteins to provide selectivity. This powerful strategy can be applied to the detection from smaller biomarkers until tissue exemplars. For example, Lee et al. (Figure 4A), developed a SERS-based sensor for quantitative detection of exosomal miRNA in serum samples of breast cancer patients [39]. Hybrid structure consists in a head-flocked gold nanopillar substrate functionalized with a specific locked nucleic acid (LNA) probes for capturing miR-21, miR-222, and miR-200c added in known concentrations to human serum. Through a sandwich strategy, LNA probes functionalized with Cy3 as Raman reporter are hybridized to further monitoring SERS peak intensity of Cy3 at 1150 cm^−1^. The closeness of gold nanoparticles creates self-assembled hotspots achieving LOD of 1 attomolar. The strategy proposed by Li et al. to detect miR-107 as biomarker for prostate cancer in urine samples consists in functionalizing both AuNPs and Au/Ag alloy nanocuboids with probes half-complementary to the whole miR-107 sequence [36]. In this way, the presence of the biomarker promotes the selective and spontaneous self-assembly of the Au spheres onto the Au/Ag nanocuboids with the specific interparticle gap (=2.3 nm) optimizing the SERS signal intensity. Indeed, inherent peroxidase-like activity of AuNPs (nanozymes) [51,52], generate catalytic cascades that oxidize TMB (3,3’,5,5’-Tetramethylbenzidine), which has a strong and recognizable SERS spectrum. Correlating TMBox SERS signal with the amount of miR-107, LOD in the range of femtomolar is achieved. These two mentioned works are innovative because they profit the assembly of plasmonic nanoparticles to create hot spots, obtaining really competitive LODs. 

However, other approaches are based on the frequency shift of the monitored peak. These approximations habitually present difficulties in terms of reproducibility, because the shifts are usually in the order of fewer than 1 cm^–1^. Zhu et al. [43] developed an Ag multiplex plasmonic substrate where Raman reports [4-mercaptobenzoic acid (MBA), 5,5‘-Dithiobis(succinimidyl-2-nitrobenzoate (DSNB) and 6-thioguanine (6TG)] were attached and functionalized against miRNA (miR-26a-5p and miR-223) and α-fetoprotein (AFP) for discriminate primary liver cancer. In the presence of the target binding, vibrational frequencies of Raman reporters are shifted. By inferring the shift in a previous calibration curve, quantification of these biomarkers can be established. Zhang et al. proposed another diagnostic approach based on frequency shift for detecting circulating tumor DNA of lung cancer in serum samples [38]. To overcome the low concentration of ctDNA (pico-to-femtomolar), a first step of enzymatic amplification of the target mediated by RNase-II was done. Amplification combined with SERS allows the detection of one single-base pair mutation on KARS G12 gene. In this case, monitoring of the Raman shift of the molecule reporter DSNB 1334 cm^−1^ peak correlates with ctDNA achieving a LOD of 1.2 x 10^−16^ M.

The improvement provided by Wang et al. consists in including magnetic properties along with the plasmonic ones, facilitating the process of separation of the analyte from the whole sample. They developed a method based on an exosomal capturing probe based on a gold shell magnetic nanostructure with antibodies against surface protein CD63 and a detecting probe consisting in AuNPs functionalized with aptamers/Raman probes (Aptamer H2/DTNB, aptamer CEA/MMC, and aptamer PSMA/2NAT), for breast cancer, colorectal cancer, and prostate cancer, respectively [41]. After adding a known concentration of capturing and detection probes to blood samples, the immunocomplex takes place, and magnetic beads are collected with a magnet. By measuring the decrease in intensity of the respective Raman reporter signal in the supernatant, quantitative analysis can be done. Moreover, Bai et al. use magnetic properties for simultaneous detection. In order to detect specific proteins for lung cancer (AFP, carcinoembryonic antigen (CEA), and ferritin (FER)) from serum, they designed a plasmonic structure codified with three different tags labeled to respective antibodies [35]. This structure will be linked through a immunosandwich assay to a magnetic bead, leading to a core-satellite structure made with both plasmonic and magnetic structures. This approximation permits a simultaneous detection with LOD of 0.15, 20, and 4 pg/mL for AFP, CEA, and FER, respectively.

Finally, the novelty provided by Lee et al. [39] consists in a dispositive able to detect two kinds of biomarkers simultaneously: CTCs and DNA. They developed a dual-function nanodevice for capturing nasopharyngeal CTCs and detecting Epstein–Barr virus (EBV) DNA from plasma samples with a LOD of 10^−13^ M [37]. For this aim, a 3D structure of Si nanowires forming microscale pyramids coated with AgNPs of 200 nm was functionalized with Anti-EpCAM antibodies to capture the above-mentioned cells and at the same time, with a probe DNA specific for EBV. EBV target DNA will be attached to a second nanostructure consisting in AgNps functionalized with 4-MBA as Raman probe. The hybridization between both probe and target DNA will cause the capturing of the second nanostructure, giving rise to the clear spectra of 4-MBA. Other approaches were developed to detect CTCs and exosomes [53]. 

### 2.3. SERS Imaging

When we move to targets like CTCs and cancer tissues, SERS can be implemented as an optical imaging technique by monitoring specific molecules. The crucial advantage in SERS imaging is the high spatial resolution of the technique as it can achieve values of < 0.5 microns in the visible range, permitting the mapping of samples with high resolution. Another advantage is also the multiplexing analysis of several analytes in the same sample. In this regard, Nima et al. synthesized 4 SEPs hybrids consisting of Au@Ag nanorod, covered with four different Raman label/antibody against breast cancer as follows: 4-MBA/anti-EpCAM, p-nitrobenzoic acid/anti-IGF-I receptor β, p-aminobenzoic acid/anti-CD44, and 4-(methylsulfanyl)thiophenol/anti-cytokeratin. After incubation of SEPs with blood containing MCF-7 cancer cells, a SERS-map of a single cancer cell was registered, obtaining the clear signal of the four Raman probes onto the cell surface [54]. Li et al., followed the same approach to multicolor imaging breast cancer cells tissues but substituting the thiolated compounds habitually used as Raman labels for alkynes and nitriles (Figure 4C) [40]. They highlight the predisposition of thiols to oxidation plus the limitation of spectral overlap. By functionalizing AuNPs of 60 nm with Tag 1 (4-ethynyl-bipheny/ER), Tag 2 (phenylthiocyanate/EGFR), and Tag 3 (4-(phenylethynyl)aniline/PR) and incubating with the tissue samples, the profiling of ER, EGFR, and PR expression in breast cancer and normal tissue sections can be done. Multiplexing approaches were used also by Bodelón et al., who employed SEPs made with gold octahedra functionalized against EGFR, EpCAM, and CD44, to detect and image one single cell in vitro of human epithelial carcinoma A431, discriminating from non-tumoral murine fibroblast 3T3 2.2 [55]. Multiplexing detection was done also in solid samples of formalin-fixed human prostate tissue by Lutz et al. By conjugating composites of organic–inorganic nanoparticles (COINs) with specific antibodies against cytokeratin-18 and PSA, a controlled aggregation of silver nanoparticles was produced, which generate images with subcellular spatial resolution [56]. Following the same rationality [57], Wang et al. developed four SEPs against cell-surface biomarkers of human breast cancer (EGFR, HER2, CD44, and CD24). The plus point of this work is the enhancement of the method by immersing the tissue surfaces into a NP-staining solution submitted to high-frequency mechanical vibration in order to promote the convection and improvement of the plasmonic materials to the biomarker targets. This approach significantly improves the speed of the ratiometric SERS-imaging. Last, an encouraging application of SERS is as a tool image endoscopy during tumor resection in order to detect minimal lesions residually left during intraoperative surgery (responsible of metastasis origin in most cases). Here, Davis et al. proposed a multiplex imaging of bladder cancer tissue made in real-time whereas transurethral resection is giving place [42]. For that aim, AuNPs functionalized with antibodies against CA9 and CD47 protein biomarkers were administrated ex-vivo in bladder cancer patients after principal surgery, to further SERS-mapping the resection margins of the tumor area in order to classify the tissue as normal or tumor, offering an in situ guide for resections. This approximation overcomes limitations of current endoscopy techniques in detecting small tumor cells, which habitually give rise to postoperative exams, high rates of re-excision, and definitively worse prognosis [58]. 

## 3. Other Techniques in Combination with SERS

**Figure 5:**
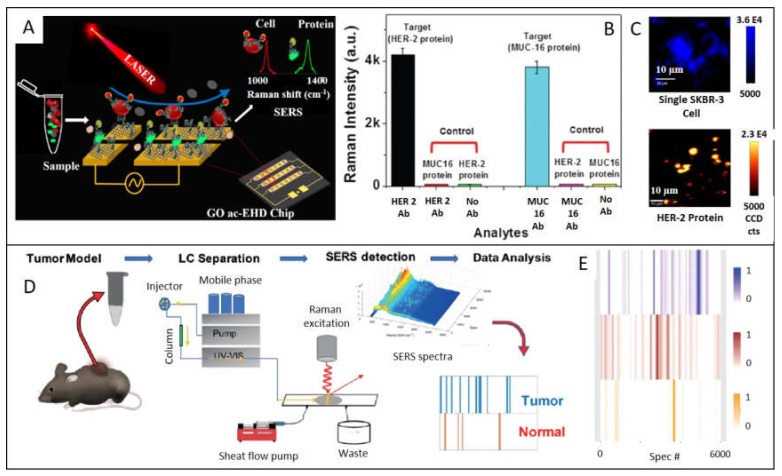
(**A**) Schematic illustration of identification of circulating protein and cell on the GO functionalized ac-EHD chip. (**B**) Device sensitivity to capture HER 2 protein (black bar) and MUC-16 (light blue bar), when they are tested through non-target proteins and blank surface (non-antibody). (**C**) SERS images of a single SKBR3 cell and HER2 proteins. Adapted with permission from [59]. Copyright Royal Society of Chemistry 2018. (**D**) Illustration of the LC-SERS based device. Metabolites extracted from mouse tumors are separated by HPLC and detected through sheath-flow SERS to make a barcode indicative of a tumor or normal tissue by collecting SERS spectra at each retention time. (**E**) Barcode of MMTV-Wnt1tumors (blue), MMTV-Neu (brown) and normal mammary gland sample (orange). Adapted with permission from [62]. Copyright Wiley 2019.

If SERS spectroscopy is a powerful tool by itself, when is combined with other techniques, possibilities are multiplied. Because of this, another interesting and prolific field of study consists in the combination of one or more techniques to take out maximum information from the less amount of sample through the less invasive methods. We bring out here the latest studies combined with SERS, as the high potential it has been demonstrated. Reza et al. developed a graphene oxide (GO) biochip, which combines alternating-current electrohydrodynamic (ac-EHD) fluid flow to simultaneously isolate CTCs and soluble protein biomarkers from biological samples, to then characterize cell surface protein biomarkers by SERS. (Figure 5A) [59]. In this manner, they profiled HER2 protein expression levels on breast cancer cell surfaces and meanwhile identified two soluble protein biomarkers (HER2 and MUC16) from simulated biological fluid. Another successful combination consists in the association of Liquid Chromatography with Surface-Enhanced Raman Spectroscopy (LC-SERS). Variation in metabolome can be indicative of suffering from a cancer disease. Current techniques in metabolomics are magnetic resonance spectroscopy (NMR) and mass spectrometry (MS), but they require high concentration of sample and challenges are present when ionizing certain metabolites in MS, limiting the scope of these techniques [60,61]. Xiao et al. centered their efforts on detecting metabolites obtained after tumor lysates by LC, to further obtain a metabolic fingerprint based on SERS spectra of the metabolites, which allow establishing patterns for classifying tumor and normal samples [62] (Figure 5 D). For that aim, metabolites extracted for MMTV-Wnt1 and C3-Tag mouse tumor models were separated by LC and submitted to a sheath-flow, which confines the eluted molecules onto an Ag nanostructured surface were SERS detector collect spectra over time. Through data analysis methods, signals can be converted into a specific barcode to identify the presence or absence of tumor. 

Fu and co-workers [63], combined the lateral flow assay strategy with SERS to detect prostate cancer PCA3 mimic DNA. Capture DNA is immobilized and hybridized in the presence of the target with DNA-AuNPs with SERS tag (MGITC). By monitoring SERS intensity, quantitative measurements can be obtained achieving a LOD of 3 fM, which outperforms available commercial kit about three orders of magnitude.

## 4. Cancer Diagnosis with Related Plasmonic Techniques

### 4.1. Surface Plasmon Resonance (SPR)

SPR technique takes advantage of the surface plasmon resonance effect in planar and thin metals, by monitoring the changes of the refractive index due to small mass variations at the plasmonic surface. In the last decades, plasmonic sensors based on this technique have emerged to detect exosomes from ovarian cancer [64], leukemia [65], lung cancer [66], tumor-specific CD8 T Cells [67], or spliced isoforms of the Fas gene [68]. Loyez et al. designed a 50 nm gold-coated optical fiber sensor functionalized with antibodies to detect the lung cancer biomarker cytokeratin 17 (CK17). By recording reflected spectra and measuring the amplitude shift in comparison with healthy tissue, a positive result of CK17 can be displayed. The innovation of this work is that it is able of test non-liquid samples, making it suitable to penetrate into soft tissues with minimally invasiveness, avoiding freeze/thaw cycles required in another biopsy technique [69]. Recently, this author developed an analogous platform to detect circulating breast cancer cells [70]. In this work, a SPR dispositive was functionalized with specific aptamers against mammaglobin-A to capture it. The novelty is the extra addition of AuNp also functionalized with the aptamers to amplify the SPR signal once they are attached to the immobilized CTC in the sensor. This device is able to detect even 10 cancer cells/mL. Several authors employ the dual SPR assisted nanoparticle-mediated amplification: Park et al. [71] to profile different transmembrane and intraexosomal proteins derived from ovarian cancer cells and Wang et al. [72] to detect exosomes derived from MCF-7 breast cancer cells. 

### 4.2. Localized Surface Plasmon Resonance (LSPR) Shift and Colorimetric Assays

Several works were developed based on variations in the LSPR peaks before and after binding to the specific targets. By following the shift in wavelength obtained after attaching of the plasmonic material (AuNPs) to the target (miR-10b), Ki et al. performed the detection of miR-10b gastric cancer-biomarker extracted from mouse blood and urine samples, with a LOD of 2.45 pM [73]. Hu et al. detected perilipin-2 (PLIN-2), a renal cell carcinoma biomarker extracted from human urine samples using Au nanorattles [74]. Na and co-workers measured miR-200a-3p in total RNA extracts from gastric cancer cell lines through a gold-based sensing platform [75]. More interesting examples can be found following this simple but efficient approach [76,77,78,79].

**Figure 6:**
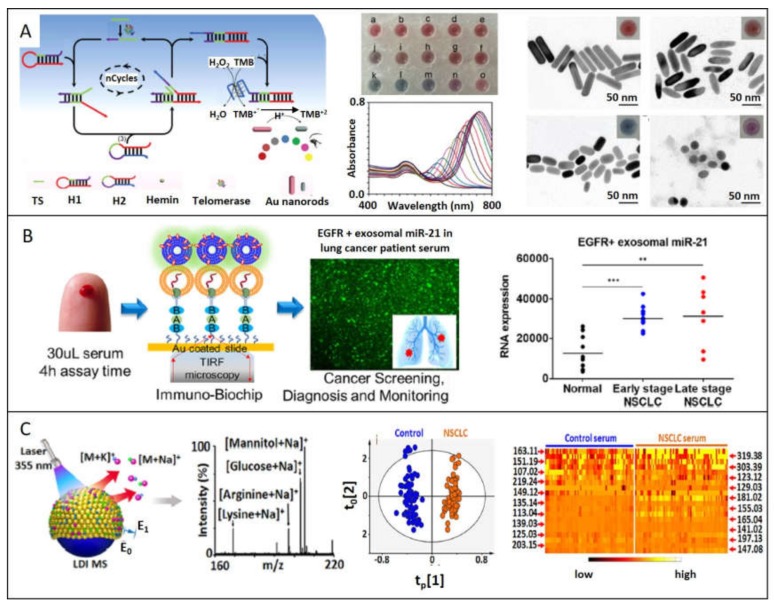
Plasmonic materials improve the detection of cancer biomarkers through several techniques: (**A**) Colorimetric assay is performed based on telomerase activity, proportional to the etching of Au nanorods. Adapted with permission from [80]. Copyright Elsevier 2019. (**B**) miR21 are effectively captured from serum sample of lung cancer patients and detected through TIRF microscopy. Adapted with permission from [90]. Copyright American Chemical Society 2019. (**C**) Clinical metabolic fingerprint obtained through laser/desorption/ionization mass spectrometry can be achieved taking advance of plasmonic materials. Adapted with permission from [91]. Copyright American Chemical Society 2018.

One study developed by Wang et al. that attracts great attention because of its simple, inexpensive, and ease-of-use performance is based on colorimetric assay monitoring of the telomerase activity [80]. This enzyme is repressed in mature somatic cells after birth, resulting in a shortening of the telomere after each cell division, but it is altered and reactivated in 85% of tumor cells, allowing uncontrolled cell proliferation [81,82]. The workflow proposed consists in a novel enzyme-free amplification method known as catalytic hairpin assembly (CHA) where the telomerase activity ends with the catalyzed H_2_O_2_-mediated oxidation of TMB. As the concentration of TMB^2+^ products increase, gold nanorods (AuNRs) present in the medium are gradually etched. This conversion can be effectively followed by the LSPR blue-shift corresponding to the decrease of aspect ratio of AuNRs, associated with a color change visible with the naked eye (Figure 6A). More authors use colorimetric assays based on plasmonic materials for monitor telomerase activity, but they principally take advantage of the cross-linking effect, which this enzyme has with AuNPs, causing aggregation and inherent red-shift LSPR peak [83,84]. As aggregation of nanoparticles is influenced by more factors, these methods are less reliable, causing false positives. Huang et al. [85] developed a colorimetric sensor for quantitative detection of breast cancer antigen 15-3 (CA15-3) through the photocatalytic property of EDTA under light irradiation, producing H_2_O_2_. EDTA-labeled antibodies against CA15-3 were assembled in a sandwich manner with antibodies immobilized onto a surface plate; for more, add HAuCl_4_. When the CA15-3 is present and EDTA-labeled antibody is attached, H_2_O_2_ byproduct from catalytic activity of EDTA can reduce gold salt to produce AuNPs. As the amount of biomarker changes, also do the AuNPs morphologies due to the influence of H_2_O_2_ in kinetics of crystal growth, obtaining different colors to correlate. If the biomarker is not present, high concentrations of hydrogen peroxide reduce gold salt to gold spheres [86]. This method was able to detect 7.5 x 10^−15^ U/mL. A similar strategy was employed by Piao et al. [87], in this case, to detect as cancer biomarker mRNA-21. EDTA-oligonucleotides were sandwich-assembled with the complementary oligonucleotides immobilized onto a SiO_2_ platform. As in the previous report, the chelating device is able to reduce Au^+3^ to form AuNPs, giving rise to colorimetric signals quantitatively and qualitatively correlated to the concentration of mRNA-21with a LOD of 8.9 femtomolar.

Following in the framework of colorimetry but moving to the area of imaging, Zhou et al. [88] developed a nanodevice to visually identify mRNA-122 of hepatocellular carcinoma through dark-field microscopy (DMF), overcoming the inconspicuous derived from small spectral LSPR shifts. With this aim, specific thiolated DNA-labeled gold nanoparticles of 50 nm in size were fabricated to recognize miRNA-122. This hybridization process changes the refractive index of AuNPs causing a red shift of the AuNPs LSPR peak, which will be gradual and proportional to the miRNA concentration. The novelty of this method resides on the selective amplification of single light scattered by the nanodevice through imaging processes techniques, improving the resolution and contrast of the DMF image and driving to a clear colorimetric scale representing the amount of cancer biomarker. Poon et al. also used dark-field microscopy for quantifying cancer biomarkers in serum, basing the amplification of the signal on the coupling of two plasmonic materials, named capture and signal-amplification probes [89]. The first one consists of 60 nm AuNPs functionalized with a primary antibody against the specific target, and the second one involves 50 nm AgNPs labeled with secondary antibody, which in presence of antigen form a sandwich immunocomplex structure. This enhancement of the intensity signal, allows the quantification of the target present in the sample. Carcinoembryonic antigen (CEA), prostate-specific antigen (PSA), and AFP were detected with this method, achieving LOD of 1.7, 3.3, and 5.9 pM, respectively.

### 4.3. Total Internal Reflection Fluorescence Microscopy (TIRF)

Plasmonic materials can also be useful in Total Internal Reflection Fluorescence microscopy (TIRF), in order to selectively excite the surface-bound fluorophores. This technique is based on the behavior of a propagating light through two media with distinct refractive indices. When light reaches a medium with a lower refractive index and is above a critical angle, it will be totally reflected with the same frequency, but this wave will decay exponentially (named evanescent field) extending around 100 nm from the interface. TIRF takes advantage of this phenomenon putting fluorophores in the near proximity of the interface, which will be excited by the evanescent field. The confinement of fluorescence emission to such a thin region results in an elevated signal-to-noise proportion. Taking advantage of this technique, Yang et al. designed a chip to selectively capture lung cancer tumor exosomes from human serum for further measure miR-21 and TTF-1 expression. (Figure 6B) [90]. By functionalizing an Au coated slide with antibodies against tumor-associated proteins (EGFR and PD-L1), absolute specificity was conquered, separating cancer exosomes from the others. In situ detection of miRNA was done through cationic lipoplexes enclosing RNA target-sensing molecular beacons (CLP-MBs), responsible for the fluorescence signal. TIRF microscopy was employed to measure MB fluorescence intensity, which correlates with the exosomal level of the target. Previous examples of plasmonic devices used in TIRF microscopy can be found to detect cancer antigen 125 [92] and AFP [93].

### 4.4. Mass Spectrometry

Sun et al. applied plasmonic properties to obtain a metabolic fingerprint from serum and exosomes of healthy and lung cancer patients by using mass spectrometry (Figure 6C) [91]. Highlighting that laser desorption/ionization (LDI) provides high sensitivity in-seconds analysis and mass measurement for molecular detection, they developed chips composed with Au nanoshells on the surface, which can be used with only 500 nL of biofluids. Small gaps and clefts of Au shells selectively catch small metabolites, which take advance of the local enhanced fields induced by the laser irradiation onto the surfaces of the nanoparticles, promoting profile analysis of complex samples. After analyzing the data through orthogonal partial least squares discriminant method, both healthy and lung cancer groups were clearly separated. 

### 4.5. Dynamic Light Scattering (DLS)

Another curious use of plasmonic nanoparticles to detect miRNA-21 with LOD of 24 pM is based on dynamic light scattering (DLS) strategy combined with strand displacement reaction (SDR) [94]. To perform this, Wang et al. designed an Au core where Au satellites were attached to the surface through a DNA linker. The presence of miRNA-21 and fuel DNA strand provokes the separation of the satellites from the core, these changes in size being optimal to be monitored by DLS. 

### 4.6. Photoacoustic Imaging

Another technique that takes advantage of plasmonic materials is photoacoustic imaging. Although is habitually less explored, it offers high temporal and spatial resolution [95]. It consists in the absorption of pulsed light to produce a short-term thermoelastic expansion, which generates ultrasonic waves susceptible to be detected and converted in an image. Indeed, plasmonic nanoparticles stand out conventional organic dyes as photoacoustic agents because they have higher extinction coefficients at LSPR wavelengths. Although photoacoustic imaging in diagnosis is usually performed in vivo [96,97,98], Ovejero et al. developed a method for isolating and detecting CTCs by combining magnetic and plasmonic properties in the same nanodevice [99]. With this aim, AuNRs and IONPs in a core-satellite disposition covered with silica were internalized into HeLa cells by incubation. Cells attached to hybrid nanodevice were easily separated to further obtain PA images using an ultrasound scanner.

## 5. Conclusions

In summary, plasmonic materials have formidable potential as bioanalytical tool to be employed in cancer diagnosis due to the fascinating optical properties at the nanoscale. As reviewed here, SERS is having an exponential growth due to exceptional advantages like small amount of sample required, narrow peak resolution, very competitive limit of detections and versatility to be used in label-free, SERS-encoded or combined methods with other techniques like liquid-chromatography. Increasingly, SERS is used in real samples, ceasing to be just proof-of-concept experiments. Even so, translation to the daily clinic is not frequently seen nowadays, although the great potential of this technique may become a reality for continuous bedside monitoring. Moreover, SPR, colorimetric assays, TIRF, mass spectrometry, DLS, and photoacoustic imaging can benefit if plasmonic properties have a promising future. 

Indeed, we have seen that several biomarkers, like proteins, exosomes, miRNA, cDNA, CTCs, and tissues can be used as specific biomarkers for fulfilling the demand of a disease as complex and multifactorial as cancer is, getting closer to personalized medicine where accurate diagnosis is crucial to an effective treatment. 

## Figures and Tables

**Table 1:** Latest insights in cancer diagnosis using Surface-enhanced Raman spectroscopy, differing among label-free and SERS-encoded particles assays.

Author	Target	Cancer	Sample	Plasmonic Material	LOD/Accuracy	Year	Reference
Koo et al.	T2:ERG ^1^, PCA3 ^2^, KLK2 ^3^ miRNA	Prostate cancer	Urine	AgNPs 40 nm	Area under curve 0.84	2018	[10]
Girish et al.	Oral squamous cell carcinoma Verrucous carcinoma Leukoplakia	Oral cancer	Mouth cancer tissue	TiO2 nanostructures covered with AgNPs 30 nm	97,24 %	2019	[32]
Carmicheal et al.	Exosome from CD18/HPAF, HPDE ^4^ and MiaPaca exosomes	Pancreatic cancer	Serum	AuNPs 10 nm onto Ti/Au 40 nm/100 nm slide	87%−90%	2019	[33]
Lin et al.	ctDNA	Nasopharyngeal cancer	Blood	AgNPs 35 nm	82.9 %	2019	[34]
Bai et al.	AFP ^5^,CEA ^6^,FER ^7^ proteins	Liver cancer	Serum	AuNPs 50 nm	0.15, 20, and 4 pg/mL	2019	[35]
Li et al.	miRNA-107	Prostate cancer	Urine	Au NSs-Hollowed alloy nanocubes	10^−15^ M	2019	[36]
Lee et al.	CTCs and EBV ^8^ DNA	Nasopharyngeal carcinoma	Blood	Si nanowires/microscale pyramids coated with Ag Nps	10^−13^ M	2019	[37]
Zhang et al.	KARS G12 mutation ctDNA	Lung cancer	Serum	Ag films	1.2 × 10^−16^ M	2019	[38]
Lee et al.	Exosomal miR-21, miR222, miR-200c	Breast cancer	Serum	Head-flocked gold nanopillar	10^−7^ M	2019	[39]
Li et al.	ER ^9^, EGFR ^10^, PR ^11^ tumor cells	Breast cancer	Cancer cells and breast cancer tissue	AuNPs 60 nm	----	2019	[40]
Wang et al.	Exosomal ErbB2 protein Exosomal PSMA ^12^ protein Exosomal CEA ^3^ protein	Breast cancer Prostate cancer Colorectal cancer	Blood	Au shell magnetic beads	32 exosomes/µL 73 exosomes/µL 203 exosomes/µL	2018	[41]
Davis et al.	CD47 and CA9 tumor proteins	Bladder Cancer	Bladder cancer tissue	AuNPs	Area under curve 0.94	2018	[42]
Zhu et al.	miR-27a-3p, miR223, miR26a-5p AFP	Liver cancer	Serum	Ag films	10^−15^ M	2018	[43]

^1^ ERG: ETS-related gene. ^2^ PCA3: Prostate cancer antigen 3. ^3^ KLK2: Kallikrein 2. ^4^ HPDE: Human pancreatic ductal epithelial cells. ^5^ AFT: Alpha-Fetoprotein Tumor. ^6^ CEA: Carcinoembryonic antigen. ^7^ FER: Ferritin. ^8^ EBV: Epstein–Barr virus. ^9^ ER: Estrogen receptor. ^10^ EGFR: Epidermal growth factor receptor. ^11^ PR: Progesteron receptor. ^12^ PSMA: Prostate-specific membrane antigen.

## Abbreviations

miRNAs	microRNAs
CTCs	Circulating tumor cells
ctDNA	Circulating tumor DNA
LSPR	Localized Surface Plasmon Resonance
SERS	Surface-enhanced Raman scattering
SPR	Surface plasmon resonance
TIRF	Total Internal Reflection Fluorescence microscopy
DLS	Dynamic light scattering
LOD	Limit of detection
SEPs	SERS-encoded particles
AuNPs	Gold nanoparticles
AgNPs	Silver nanoparticles
